# Physical Modification of Whey Protein by Interacting with Methyl Hesperidin: Impacts on Antioxidant Activity and Underlying Mechanism

**DOI:** 10.3390/biology14050492

**Published:** 2025-05-01

**Authors:** Songbo Ma, Shuang Yang, Suqi Yang, Lili Chen, Meilan Yuan, Yong Jiang, Li Zhao, Chunqing Bai

**Affiliations:** National R&D Branch Center for Freshwater Fish Processing, College of Life Science, Jiangxi Science and Technology Normal University, Nanchang 330013, China; msb15838343158@163.com (S.M.); z13197945118@163.com (S.Y.); b20040461@163.com (S.Y.); 1020140971@jxstnu.edu.cn (L.C.); 1020100975@jxstnu.edu.cn (M.Y.); 1020130968@jxstnu.edu.cn (Y.J.)

**Keywords:** methyl hesperidin, whey protein, antioxidative activity, interaction, molecular docking

## Abstract

In this work, the possibility of enhancing the antioxidant capacity of whey protein (WP) through non-covalent interaction with methyl hesperidin (MH, a hesperidin derivative) was assessed. The underlying mechanism was analyzed in terms of multi-spectroscopy methods, thermodynamic analysis, and molecular docking simulation. The data indicated that MH could spontaneously bind to WP and form a non-fluorescent complex when physically mixed together. The presence of MH statically quenched the intrinsic fluorescence of WP, changed the microenvironment of amino acid residue, and altered the secondary and tertiary structure of WP, which in turn enhanced the antioxidant capacity of WP. The underlying mechanism may be assigned to hydrophobic interactions, which promoted MH inserting itself into the hydrophobic cavity in WP. The methoxy group on the B ring of MH may form hydrogen bonds with amino acids, which enhances the freedom of the phenyl hydroxyl group, resulting in higher antioxidant capacity than other hesperidin structural analogs. This research would enrich the theoretical basis about the interaction between protein and hesperidin-based derivatives, and it may supply valuable information for its application in the food and medicine fields.

## Simple Summary

Whey protein is a widely utilized food ingredient known for its favorable nutritional profile and functional properties. Nevertheless, its inherent antioxidant capacity is relatively limited, which restricts its application as a natural antioxidant in the food industry. This study aims to investigate an environmentally friendly method to enhance the antioxidant activity of whey protein. In this process, a natural flavonoid derivative, methyl hesperidin, was introduced into a whey protein solution. Through physical mixing, it was observed that the two components could spontaneously bind to form a non-covalent complex. Furthermore, the microenvironment of amino acid residues in whey protein was altered with changed conformation, leading to enhancement in its antioxidant capacity. Additionally, molecular docking studies revealed that methyl hesperidin inserted itself into the hydrophobic cavity of the whey protein. These findings not only propose a novel approach for developing healthier antioxidants but also contribute to a deeper understanding of the interaction mechanisms between proteins and flavonoids.

## 1. Introduction

Whey protein (WP), as an important nutrient in milk, has attracted great attention from consumers, researchers, and industries due to its nutritional profile and functional properties [1]. The protein has been reported to contain abundant essential amino acids and to promote human health and growth [2]. Moreover, it has been proven to exhibit excellent emulsifying, forming, and gelling properties and has been widely used as an emulsifier and encapsulating agent in the food industry [3]. However, like many other proteins, WP has relatively low antioxidant activity, which has confined its utilization as a natural antioxidant in the food industries, or else it must be added with other antioxidants to achieve food oxidation.

Fortunately, the problem has been reported to be overcome through structural modifications [4]. Physical, chemical, and biological modifications can often be used for such purposes. Compared to the latter two methods, physical modification is usually more cost-effective and does not require regulatory approval [5,6]. The method has become increasingly popular in recent years [7]. During physical modification, antioxidant molecules are attached to the surface of the protein. The suit antioxidants usually play an important role in the binding interactions, which further affects the property of the modified protein [8].

Phenolic compounds, as natural antioxidants generated in plants, are good candidates. Generally, there is at least one phenolic hydroxyl group within their molecules [9,10], which endows the phenolic compounds with the excellent antioxidant ability to scavenge free radicals. Moreover, phenolic hydroxyl groups are very inactive and are prone to spontaneously interacting with proteins and binding together, which changes the proteins’ conformation and alters their functional properties. The formed phenolic-protein complexes could be utilized as multi-functional food additives, i.e., antioxidant gelatos [11]. As a result, increasingly more researchers have devoted themselves to investigating the interaction and structural functional effects of polyphenols on proteins [8,9,12,13]. The interactions between common dietary polyphenols (chlorogenic acid, epicatechins, gallic acid, tannins, caffeic acid, etc.) and proteins (gelatin, soy protein, casein, peanut protein, rice protein, etc.) have been intensively investigated in recent years. Jiang et al. assessed the effects of chlorogenic acid binding on the solubility, structure, and foaming ability of casein and whey protein [14]. Dai et al. found that the interaction of proanthocyanidins with rice proteins enhanced rice proteins’ surface hydrophobicity [15]. Xu et al. indicated that the antioxidant ability of the whey protein-chlorogenic acid complex was significantly increased after the covalent binding of chlorogenic acid to whey protein [16].

**Figure 1:**
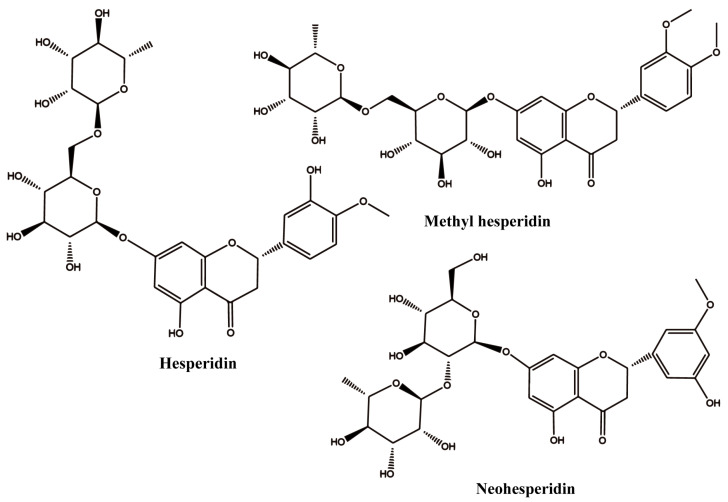
The 2D structures of hesperidin, methyl hesperidin, and neohesperidin.

Hesperidin (HES) is a flavonoid polyphenol compound extracted from Rutaceae plants, such as bergamot, kumquat, lemon, citron, and shepherd’s purse. It has been used by some researchers to modify proteins due to its excellent anti-inflammatory and antioxidant properties. Wang et al. reported that hesperidin could bind to whey protein and form a complex with it, which could improve the antioxidant activity of the whey protein [17]. Jiang et al. found that binding with hesperidin could enhance the thermal stability and emulsification activity of gluten [10]. However, its water solubility is very limited, such that hesperidin must be dissolved in ethanol or methanol before being mixed with protein, which significantly limits its wide application [18]. To address this problem, researchers have engaged in the synthesis of various hesperidin-based derivatives, including MH, neohesperidin (NH), etc. Their structure is shown in Figure 1.

Among them, MH is a notable derivative. By introducing a methoxy group into the B ring, the obtained methyl hesperidin exhibits good water solubility and permeability compared to hesperidin [19]. However, information involving the interaction between MH and protein is very limited. The only information that can be found indicates that MH could bind to serum albumin and change its conformation [20]. However, whether MH could interact with whey protein and whether the introduction of methyl hesperidin can improve the unsatisfactory antioxidant activity of whey protein is still unknown.

Therefore, the aims of this research were to explore the possible interaction between MH and WP. Fluorescence spectroscopy, synchronous fluorescence spectroscopy, circular dichroism (CD) spectra, and thermodynamic analysis combined with molecular docking simulation were applied to predict the underlying mechanism. The antioxidant capacity of WP in the presence of MH was evaluated in terms of 2,2′-Azinobis-(3-ethylbenzothiazoline-6-sulfonate) (ABTS) assay, 1,1-diphenyl-2-picrylhydrazyl radical (DPPH) assay, and ferric ion-reducing antioxidant power (FRAP) assay. This research provides information for improving the functional activity of protein using MH, which can offer good references for the further application of the WP-MH complex in food and medicine products.

## 2. Materials and Methods

### 2.1. Materials and Chemical

WP (purity > 90%) was acquired from Shanghai Yuanye Bio-Technology Co., Ltd. (Shanghai, China). MH (purity > 90%), DPPH, 2,4,6-Tri(2-pyridyl)-s-triazine (TPTZ), and ABTS were obtained from Shanghai Aladdin Biochemical Technology Co., Ltd. (Shanghai, China). Disodium hydrogen phosphate, anhydrous ethanol, ferric chloride, sodium acetate, glacial acetic acid, and sodium dihydrogen phosphate were purchased from Xilong Scientific Co., Ltd. (Shantou, China). All other chemical reagents used in this research were of analytical grade. Ultrapure water was utilized throughout the research. Furthermore, a 0.5 mg/mL WP stock solution was pre-prepared by dissolving it in a 0.01 M phosphate buffer saline (PBS, pH 6.8) and then diluting it to 0.1 mg/mL before experimentation. In addition, 1 × 10^−3^ mol/L of MH stock solution in ultrapure water was also prepared and stored at 4 °C for further use.

### 2.2. Fluorescence Spectra

A series of MH-WPI complexes containing different amounts of MH (0–26.7μM) were prepared according to previous methods with slight modification [21]. In brief, different volumes (0, 20, 30, 40, 50, 60, 70, and 80 μL) of MH solutions were added into 3.0 mL of WP solutions fixed at 0.1 mg/mL. After thoroughly mixing for 2 h at different temperatures (298 K, 304 K, 310 K), the obtained complexes were transferred into quartz cuvettes. The fluorescence spectra of these samples were measured using an FL2700 fluorescence spectrophotometer (Hitachi, Tokyo, Japan) by setting the excitation wavelength to 280 nm and excitation and emission slit widths to 5 nm. The emission spectra were recorded in a wavelength range of 300–500 nm.

### 2.3. Synchronous Fluorescence Spectrometry

The synchronous fluorescence spectra of the complexes that were incubated at 298 K were characterized in the range of 200–350 nm. The excitation and emission wavelength interval (Δλ) settings at 15 nm and 60 nm were utilized to investigate the spectroscopic characteristics of tyrosine (Tyr) and tryptophan (Trp) residues in WP.

### 2.4. Circular Dichroism (CD) Spectra Measurements

The CD spectra of WP, both in the presence and absence of MH, were conducted on a Chirascan V100 CD spectrometer (Applied Photophysics, Surrey, UK). The instrument parameters were set with a slit bandwidth of 1 nm, a scanning rate of 100 nm/min, and a time constant of 0.5 s. For all samples, the concentration of WP was maintained at 0.385 mg/mL. The solution was mixed with different concentrations of MH (the mass ratios of WP to MH were 1:0, 2:1, 1:1, 1:2, and 1:4), incubated at 25 °C for 2 h, and then determined in a wavelength range of 190–260 nm. The contents of the secondary structures of these mixtures were calculated using CDNN 2.1 software (Applied Photophysics, Surrey, UK).

### 2.5. Molecular Docking

#### 2.5.1. Receptor and Ligand Preparation

α-Lactalbumin (α-LA) and β-Lactoglobulin (β-LG) are the main components of WP, the crystal structures (PDB ID: 1F6S and ID: 1BEB) of which were obtained from the Protein Data Bank (https://www.rcsb.org/ accessed on 9 November 2024). The model structure for MH (ID: 5284419) was retrieved from the PubChem database (https://pubchem.ncbi.nlm.nih.gov/ accessed on 9 November 2024). The water molecules and ligands present in α-LA and β-LG were removed using the PyMOL tool prior to performing the simulations. Hydrogen atoms and charges were subsequently integrated into the protein structures using AutoDock Tools (The Scripps Research Institute, San Diego, CA, USA). For MH, the MM2 force field under Chem3D software (version 20.0) was used for energy minimization to obtain a stable 3D conformation. The optimized MH structure was protonated in Tools. The processed pdb files were converted to pdbqt files waiting for docking, respectively.

#### 2.5.2. Docking Method

The AutoDock Vina Program (version 1.5.7) was employed to dock the binding process of MH to WP [22]. This methodology refers to the previous one with appropriate modifications [23]. α-Lactalbumin (α-LA) and β-Lactoglobulin (β-LG) are the main components of WP, the crystal structures (PDB ID: 1F6S and ID: 1BEB) of which were obtained from the Protein Data Bank (https://www.rcsb.org/ accessed on 9 November 2024). The model structure for MH (ID: 5284419) was retrieved from the PubChem database (https://pubchem.ncbi.nlm.nih.gov/ accessed on 9 November 2024). Before conducting the simulations, the water molecules and ligands present in α-LA and β-LG were removed by utilizing PyMOL tools. Hydrogen atoms and charges were subsequently incorporated into the protein structures prior to the simulation. In the docking process, the ligand (MH) remained flexible, while the receptors (α-LA/β-LG) were constrained. The center coordinates of 1F6S were set at 42.051, 93.085, and 10.657, and those of 1BEB were −12.765, 15.631, and −9.562. The size of the docking boxes was 50 Å × 28 Å × 37 Å and 45 Å × 42 Å × 42 Å for 1F6S and 1BEB, respectively. The top 20 pose docking results were saved, and the one that had a smaller energy and better percentage frequency could be selected as the best. Finally, the interaction between MH and α-LA/β-LG was visualized using PyMOL (DeLano Scientific LLC, South San Francisco, CA, USA) [24] and Discovery Studio 2019 software (Dassault Systèmes, Concord, MA, USA) [25].

### 2.6. Determination of Antioxidant Activity

The antioxidant capacity of WP-MH complexes was evaluated using three different methods: ABTS, DPPH, and FRAP assays. The antioxidant activity of pure WP and free MH was also determined for comparison.

#### 2.6.1. ABTS Assay

The ABTS free radical scavenging ability of free MH, pure WP, and MH-WP complexes was measured according to a previously reported method [26]. In brief, 1.5 mL of the samples mixed with 0.5 mL of ABTS+ working reagent were stored in dark conditions for 6 min and then measured at 734 nm on a UV-Visible spectrophotometer (U-T6A, Shanghai Yipuyiqi Co., Ltd., Shanghai, China). The absorbance of the mixtures was recorded as A_1_. The absorbance of the control group (A_0_) was operated using the same procedure, except using PBS buffer solution instead of the sample to react with the ABTS working reagent. All measurements were repeated at least three times. The ABTS free radical scavenging rate was calculated using Equation (1). The antioxidant capacity was expressed as the equivalent Trolox (TEAC).

ABTS free radical rate (%) = (A_0_ − A_1_)/A_0_ × 100 (1)

#### 2.6.2. DPPH Assay

The DPPH free radical scavenging ability of all samples was determined according to Wu et al. [27]. In brief, 2 mL of the sample was mixed with 1 mL of the DPPH working reagent, incubated in a black environment for 30 min, and then determined at 517 nm (A_t_). The blank sample and control sample were also determined according to the same procedure, except that absolute ethanol was used instead of the sample to mix with the DPPH working reagent (A_b_) and absolute ethanol was used instead of DPPH (A_c_), respectively. The capacity of scavenging DPPH free radicals was calculated using Equation (2) and expressed as TEAC.

DPPH free radical rate (%) = (A_b_ − A_t_ − A_c_)/A_b_ × 100 (2)

#### 2.6.3. FEAP Assay

The ferric-reducing power of the samples was determined as previously described [28], with slight modifications. Then, 1 mL samples were combined with 4.5 mL of the TPTZ working reagent and incubated in a dark environment for 10 min. The absorbance of the mixtures was then measured at 593 nm on a UV spectrophotometer and recorded as A_t_. The blank group (A_0_) and the control group (A_1_) were also determined following the same method. For the blank, deionized water was used to react with the TPTZ solution instead of the sample, while for the control, deionized water was used instead of the TPTZ solution to react with the sample. The ferric-reducing power of each sample was calculated using Equation (3).

Ferric-reducing power = A_t_ − A_1_ − A_0_ (3)

### 2.7. Statistical Analysis

The data were expressed as the means ± standard deviation (SD) based on the measurement carried out in triplicate under consistent conditions. Graphs were generated using Origin software 2019 version. The statistical significance was analyzed using one-way ANOVA with Duncan’s test, which was conducted on IBM SPSS statistics software (version 26), with *p* < 0.05 indicating statistical significance.

## 3. Results and Discussion

### 3.1. Fluorescence Spectrum

**Figure 2:**
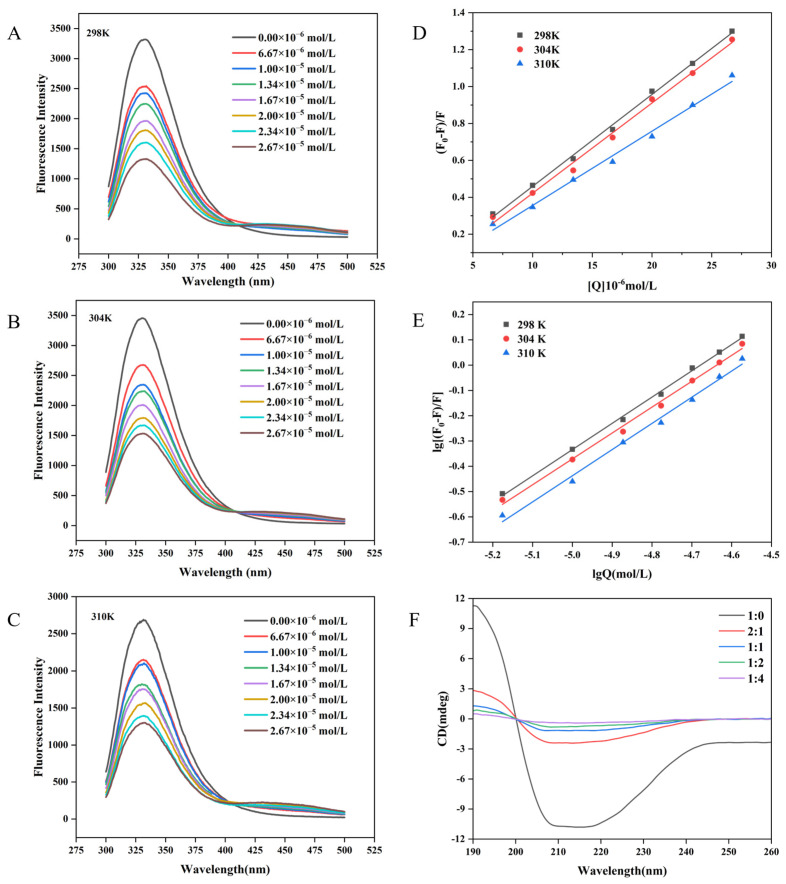
Fluorescence spectra of WP in the presence of different concentrations of MH at 298 K (**A**), 304 K (**B**), and 310 K (**C**). The Stern-Volmer plots (**D**) and double-log plots (**E**) for the fluorescence quenching of WP by MH at different temperatures. CD spectral of WP in the presence of MH at 298 K (**F**). In the figure, F and F_0_ represent the fluorescence intensity of WP before and after MH addition, respectively, and Q is the concentration of MH.

Fluorescence is an important tool for investigating conformational changes in proteins and could provide insight into the binding between polyphenols and proteins, as well as the microenvironmental changes in the amino acid residues of proteins [29]. In general, the endogenous fluorescence of proteins is mainly attributed to tryptophan (Trp), tyrosine (Tyr), and phenylalanine (Phe) residues, which contain benzene rings or conjugated double bonds in their molecular structure and could emit fluorescence at specific wavelengths [30]. The absorbance peaks for Trp, Tyr, and Phe are often located around 348, 303, and 282 nm, respectively. Compared to others, the fluorescence of Phe residues in protein is relatively weak and is prone to rapid quenching. Consequently, the fluorescence spectroscopy of the proteins detected is primarily contributed by Trp and Tyr residues [31,32]. As shown in Figure 2, the maximum fluorescence emission of WP appeared at around 345 nm when excited at 280 nm, indicating that the intrinsic WPI fluorophore was mainly generated by tryptophan residue. This was consistent with Wang et al. [17] and Ma et al. [33]. Additionally, MH exhibited a weak fluorescence emission signal in the preliminary experiment, suggesting the fluorescence signal of WP was not significantly interfered with by MH. Therefore, there was no need to consider the interference of the fluorescence inner filter effect [34].

To explore the interaction between WP and MH, the fluorescence emission spectra of WP in the presence of different concentrations of MH (0–26.7 μM) at different temperatures were further determined. As illustrated in Figure 2A–C, although the shape of the fluorescence emission was unchanged after introducing MH, the fluorescence intensity decreased gradually with a slight red shift (2 nm) in the maximum fluorescence emission as the MH concentration was increased. These phenomena indicated that the intensity and wavelength of the intrinsic WP fluorescence peak were sensitive to MH, which may interact with WP and form a non-fluorescence complex, leading to the quenched intrinsic WP fluorescence. The redshift also suggested that the introduced MH transferred tryptophan residues in WP toward the hydrophilic microenvironment and altered the WP conformation [35]. Similar results were also reported in previous studies. He et al. [36] found that malvidin-3-O-glucoside could quench milk α- and β-casein intrinsic fluorescence and induce a 3 nm blue shift in the maximum peak. Y. Shu et al. noted that the addition of erucic acid could bind to bovine serum albumin and induce conformational changes, leading to the quenching of intrinsic fluorescence and a decrease in fluorescence intensity in bovine serum albumin [37]. Wang et al. reported that the intrinsic WP fluorescence intensity was diminished in the presence of hesperidin, with the maximum peak shifted to higher numbers (2 nm) [17]. Similar results were also found in the interaction between neohesperidin and WP [33].

### 3.2. Fluorescence Quenching Mechanism

To explore the interaction mechanism of WP with MH, the fluorescence quenching behavior of WP with MH was subsequently analyzed using the Stern-Volmer Equation (4). The fitted curves and relevant parameters are summarized in Figure 2D and Table 1, respectively. Notably, all fitted curves displayed a good linear relationship with the MH concentrations, suggesting that the interaction between MH and WP was predominantly attributed to static quenching or dynamic quenching [38]. In general, static quenching was characterized by forming non-fluorescent complexes between the fluorophore and the quencher, leading to reduced fluorescence intensity. That means the quenching constant decreased with the increase in temperature. Whereas for dynamic quenching, it was the collisions between the fluorophore and the quencher that induced the decreased fluorescence intensity. Higher temperature means more chances of collisions and a higher quenching constant [39]. To ascertain which quenching mechanism is more fitted for the interaction between MH and WP, the obtained quenching constant also needed to be compared with the maximum dynamic quenching constant (2.0 × 10^10^ L·mol^−1^·s^−1^). If the fluorescence quenching constant is higher than the maximum dynamic quenching constant, static quenching is the more suitable mechanism. On the contrary, if K_q_ is smaller than 2.0 × 10^10^ L·mol^−1^·s^−1^, dynamic quenching is more fitted [40]. As illustrated in Table 1, the K_sv_ value gradually decreased as the temperature increased; it was 0.50, 0.49, and 0.40 × 10^5^ L·mol^−1^ at 298 K, 304 K, and 310 K, respectively, with the smallest K_q_ value (4.02 × 10^12^ L·mol^−1^·s^−1^) being significantly higher than 2.0 × 10^10^ L·mol^−1^·s^−1^, indicating that MH could statically quench the intrinsic WP fluorophore. Furthermore, the decreased K_sv_ value at higher temperatures suggests that it was difficult for MH to form a complex with WP at higher temperatures [41].

To determine the binding constant (K_a_) and the binding site number (*n*) for MH to WP, a double logarithmic Equation (5) was employed to calculate the static quenching. As shown in Table 1, the n values were all close to 1, implying that WP has one binding site with MH. In addition, the trend of the K_a_ value versus temperature was similar to that of the K_sv_ value, suggesting that the binding force became weaker as the temperature increased [21,42], which may generate unstable complexes [10,41]. In our previous work, the quenching mechanism of hesperidin to WP was investigated. Although hesperidin could statically quench the endogenous fluorescence of WP, the binding constants and quenching constant of hesperidin were lower than those of MH at fixed temperatures (304 K and 310 K). For example, the K_sv_ and K_a_ for MH were 0.49 × 10^5^ M^−1^ and 5.56 × 10^4^ M^−1^ (at 304 K), respectively, whereas they were 0.39 × 10^5^ M^−1^ and 3.89 × 10^4^ M^−1^ for hesperidin, respectively. What is more, the negative temperature influence on the stability of the formed complex was more remarkable for hesperidin than for MH. The difference may be assigned to one more methoxyl group in MH’s B ring, which might promote the formation of more hydrogen bonds between MH and WP, which helped to maintain the interaction between them [17].

F_0_/F = 1 + K_q_τ_0_[Q] = 1 + K_sv_[Q] (4)

lg [(F_0_ − F)/F] = lg K_a_ + n lg[Q] (5)

Here, F_0_ and F represent the fluorescent intensity of WP before and after the introduction of MH, respectively. τ_0_, generally 10^−8^ s, [Q] is the molar concentration of MH. K_sv_ represents the quenching constant for the Stern-Volmer curves. K_q_ is the quenching rate parameter, K_a_ symbolizes the binding constant, and n is the number of binding sites.

### 3.3. Thermodynamic Parameters

To better understand the main forces driving the interaction between MH and WP, the Van’t Hoff equation (Equation (6)) and free energy equation (Equation (7)) were then applied to calculate the thermodynamic parameters, including enthalpy change (ΔH), entropy change (ΔS), and free energy change (ΔG).

ln K_a_ = −ΔH/RT + ΔS/R (6)

ΔG = ΔH − TΔS(7)

Here, R represents the gas constant, which is equal to 8.314 J·mol^−1^·K^−1^. T is the absolute temperature. Ka is equivalent to the one displayed in Equation (5). The ΔH and ΔS values were derived from the slope and intercept of the linear Van’t Hoff curves, which were generated by plotting ln K_a_ against 1/T. Equation (7) was employed to calculate ΔG.

Previous reports implied that there are mainly four types of binding forces involved in the formation of complexes. They are hydrogen bonding, hydrophobic interactions, van der Waals forces, and electrostatic forces [43,44]. What is more, the specific binding force between proteins and small molecules could be deduced through analyzing the magnitudes of thermodynamic parameters. Positive ΔH and ΔS values suggest that hydrophobic interactions are the predominant force in the complex, while positive ΔS and negative ΔH values indicate that electrostatic forces play a key role. Negative ΔS and positive ΔH values suggest that electrostatic forces and hydrophobic interactions are the driving forces. Conversely, both ΔH and ΔS negative values indicate that van der Waals forces and hydrogen bonding interactions are predominant factors [45].

As shown in Table 2, both ΔH and ΔS for WP-MH were positive (25.40 KJ/mol and 0.17 KJ/mol/K, respectively), suggesting that hydrophobic interactions were the primary driving force for the MH and WP complex. Furthermore, the negative ΔG indicated that the combination of MH and WP was spontaneous [46]. Moreover, the ΔH value (25.40 KJ/mol) was positive, indicating that the binding of MH and WP was an endothermic process. This discovery aligns with prior studies, which found that hydrophobic interactions were a primary force for the interaction between flavonoids and proteins, such as bovine serum albumin [47], rice protein [15], and β-lactoglobulin [36]. In addition, the positive ΔH value also implies that higher temperatures would favor the endothermic reaction. However, the generated unstable complex at higher temperatures may be due to fact that the hydrophobic interactions between MH and WP were weaker at higher temperatures [21]. This could provide good explanations for the changes in K_sv_ and K_a_ as a function of temperature for MH and hesperidin.

### 3.4. Synchronous Fluorescence

**Figure 3:**
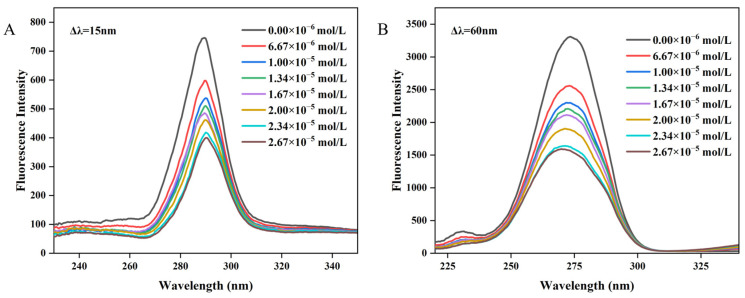
The synchronous spectroscopy of WP interacting with MH. (**A**) Δλ = 15 nm, (**B**) Δλ = 60 nm.

Synchronous fluorescence spectroscopy serves as a valuable technique for elucidating the alterations in the local microenvironment of amino acid residues within proteins and for assessing the effects of polyphenol binding on protein conformation [48,49]. Specifically, by setting Δλ at 60 nm and Δλ = 15 nm, the resultant synchronous fluorescence spectra could provide detailed information regarding the spectral characteristics of the microenvironments surrounding tryptophan (Trp) and tyrosine (Tyr) residues in proteins, respectively [42,50]. The synchronous fluorescence spectra of Tyr and Trp residues in WP at varying concentrations of MH are depicted in Figure 3A,B. Notably, the fluorescence intensity of Trp was significantly higher than that of Tyr, indicating that Trp residues were the main contributors to the endogenous fluorescence of WP. As the concentration of MH increased, the fluorescence intensity of both amino acid residues exhibited a gradual decline. For instance, at an MH concentration of 2.67 × 10^−5^ mol/L, the fluorescence intensity of Trp decreased by 51.92%, from 3311 to 1592, while the fluorescence intensity of Tyr decreased by 46.38%, from 746 to 400. The more pronounced reduction in Trp fluorescence intensity may be attributed to its closer proximity to the binding site, suggesting a greater contribution in the binding process compared to Tyr [51]. The observed changes in synchronous spectroscopy together with the fluorescence spectrum suggest the formation of a non-fluorescent complex between MH and WP [52]. According to the literature, the shift in the maximum emission wavelength position can reveal alterations in the microenvironment surrounding Trp or Tyr residues [53]. As shown in Figure 3B, when Δλ = 60 nm, a blue shift from 274 nm to 270 nm was observed in the maximum wavelength, indicating an increase in the hydrophobicity or a decrease in the polarity of the tryptophan microenvironment after MH-WP binding [54,55]. Conversely, the maximum emission wavelength of Tyr remained unchanged. Overall, the altered microenvironment of Tyr and Trp residues suggested the conformation changed for WP after binding with MH.

### 3.5. Circular Dichroism Spectra

Circular dichroism (CD) was effective in assessing alterations in the secondary structure of proteins. Figure 2F illustrated the influence of MH concentrations on the spectrum of WP. The corresponding content of the secondary structure is summarized in Table 3. Notably, WP exhibited two negative bands located at approximately 208 nm and 218 nm, which were the characteristic peaks of the α-helix and β-sheet structure, respectively [56]. However, the bands shifted upwards in the presence of MH, and more MH resulted in greater shifts. Instances in the literature have reported that a downward shift was related to increased helical structure, whereas an upward movement in the spectrum meant that the helical structure was decreased [57]. Consequently, MH may interact with WP and deduce the helical structure of WP. The finding could also be certified by the changes in its secondary structure composition. As shown in Table 3, the content of α-helix was 18.8%, which decreased to 6.4% when the weight ratio of WP to MH reached 1:4. Meanwhile, other secondary structures also underwent various changes: slight decreases were observed for β-turn (from 19.2% to 16.7%) and random coiling (30.5% to 28.8%), whereas a significant increment (31.1% to 52.9%) was monitored in the content of β-sheet.

According to instances in the literature, the stability of the α-helix mainly depends on the hydrogen bonds in the peptide, while the formation of the β-sheet partially depends on the interactions between hydrophobic groups besides hydrogen bonds [58]. As illustrated in the molecular docking simulation section, MH entered into the hydrophobic cavity of WP and interacted with the amino acids there. The interaction may disrupt the hydrogen bonds that were originally used to maintain the helix structure, leading to the instability of the structure and subsequently causing helicolysis, which resulted in a decreased α-helix content in the end. During the dissociation of the α-helix, the broken hydrogen bonds may partly rearrange and form new hydrogen bonds, which promoted the stabilization of the β-sheet structure. Meanwhile, as stated in Section 3.4, the binding of MH induced some residue exposure of hydrophobic groups of amino acids and enhanced the hydrophobicity of WP, which may further promote the formation of more β-sheet structures. Generally speaking, α-helix, as a type of high-tight structure, is mainly located in the inner polypeptide chains. As compared to the α-helix, the structure of the β-sheet is more flexible and looser. In this sense, the significant changes in the content of the β-sheet and a-helix may be assigned to MH, which entered into the hydrophobic cavity of WP and destroyed the ordered structures, turning them into unordered and loose ones. These results are consistent with previous research by Dai et al., who found that resveratrol could intercalate into the hydrophobic region of rice protein, disrupting its hydrogen bond network and subsequently reducing the α-helix content [15]. Additionally, Tang et al. demonstrated that the addition of cyanidin-3-glucoside (C3G) could loosen the structures of hemoglobin and myoglobin [59]. Similar results were also found by Zhang et al., who further confirmed that the enhancement of protein emulsification and water solubility were attributed to the transformed disordered state [60]. In this sense, the alteration in the secondary structure induced by MH may play an important role in the enhanced antioxidant activity of WP. However, the specific mechanism for the decrease in β-turn and random coiling was very unclear. It may be due to the non-covalent interactions between MH and WP, which weaken the solvation between WP and water molecules, causing the side chains of WP to fold more tightly [59].

### 3.6. Molecular Docking Simulation

**Figure 4:**
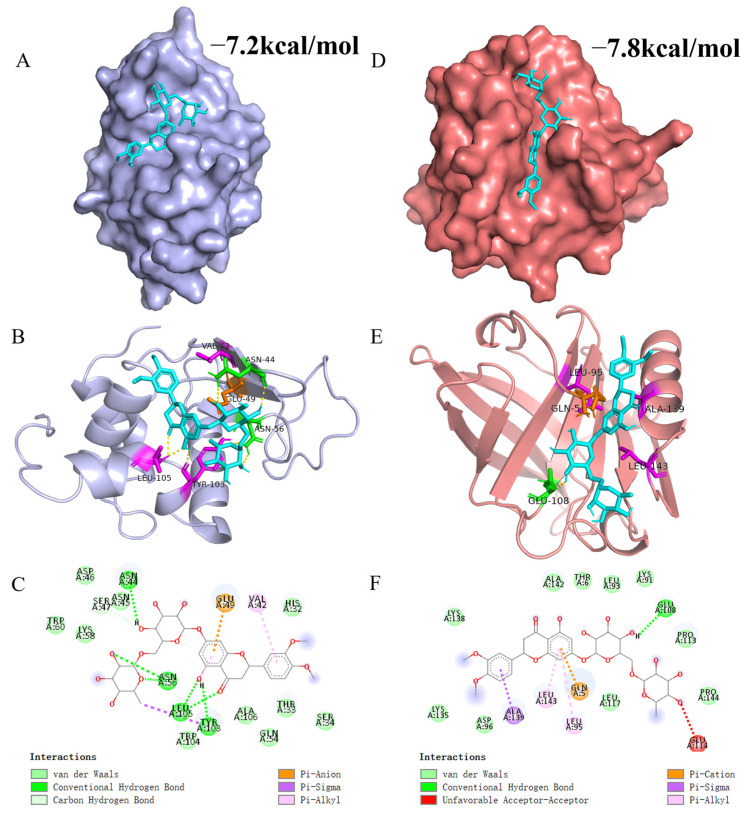
3D and 2D figures illustrating the interaction between MH and WP. (**A**,**D**) represent surface models of the interaction between MH and α-LA/β-LG. (**B**,**E**) represent 3D plots of the interaction between MH and α-LA/β-LG. (**C**,**F**) display 2D plots of the interaction between MH and α-LA/β-LG. In these figures, MH is represented by cyan, while green, purple, and orange represent hydrogen bonding, hydrophobic interactions, and electrostatic interactions, respectively.

Molecular docking simulations are highly valuable for gaining an in-depth understanding of the interactions between receptor and ligand molecules. In this research, MH was utilized as the ligand, and α-LA or β-LG, which are the predominant ingredients in WP, were chosen as the receptor. Figure 4 displays the most probable molecular interaction conformation between α-LA/β-LG and MH (selected based on their lowest energy scores; the affinities were −7.2 kcal/mol and −7.8 kcal/mol, respectively).

The 3D and 2D interaction results in Figure 4 reveal that MH inserts itself into a hydrophobic cavity in α-LA. Specifically, MH is surrounded by 17 amino acid residues in α-LA, including His32, Thr33, Ser34, Val42, Asn44, Asn45, Asp46, Ser47, Glu49, Gln54, Asn56, Lys58, Trp60, Tyr103, Trp104, Leu105, and Ala106. Among these residues, Asn44, Ser47, Tyr103, and Leu105 formed seven hydrogen bonds with MH. Additionally, the benzene ring of MH engaged in pi-Alkyl interactions with Val42 and Leu105, while a Pi-Sigma hydrophobic interaction occurred between the aromatic ring of Tyr103 and MH. An electrostatic interaction (Pi-Anion) was observed between MH and Glu49. Thus, the main driving forces that facilitated the interaction between MH and α-LA were Van der Waals forces, hydrophobic interaction, and hydrogen bonds. Similarly, MH entered into the hydrophobic cavity of β-LG and was surrounded by Gln5, Thr6, Lys91, Leu93, Leu95, Asp96, Glu108, Pro113, Glu114, Leu117, Lys135, Lys138, Ala139, Ala142, Leu143, and Pro144. In β-LG, the benzene ring of MH mainly formed three hydrophobic interactions (two pi-Alkyl and one Pi-Sigma) with Leu95, Ala139, and Leu143. Furthermore, a hydrogen bond could be formed through the hydrogen atom of MH with Glu108 of β-LG. Moreover, the benzene ring of MH also formed a Pi-Cation interaction with Gln5. The difference in binding strength between MH and α-LA or β-LG is primarily attributed to their distinct structural differences. Evidently, the β-LG crystal might be more suitable for simulating the interaction between MH and WP because the simulated results were more consistent with the thermodynamic analysis. This finding aligns with reports from other researchers who highlighted the significant role of hydrophobic interactions in maintaining the interaction between proteins and polyphenolic compounds. Wang et al. found that hydrophobic interactions were the primary forces in the binding interaction of WP and hesperidin, with additional contributions from hydrogen bonds and van der Waals forces [17]. Khalifa et al. reported that the WP-anthocynins complex was predominantly stabilized through hydrophobic interactions [61].

### 3.7. Antioxidant Assay

In this section, the effect of MH on the antioxidant capacities of WP was evaluated using ABTS assay, DPPH assay, and FRAP reducing power.

#### 3.7.1. ABTS Free Radical Scavenging Capacity

**Figure 5:**
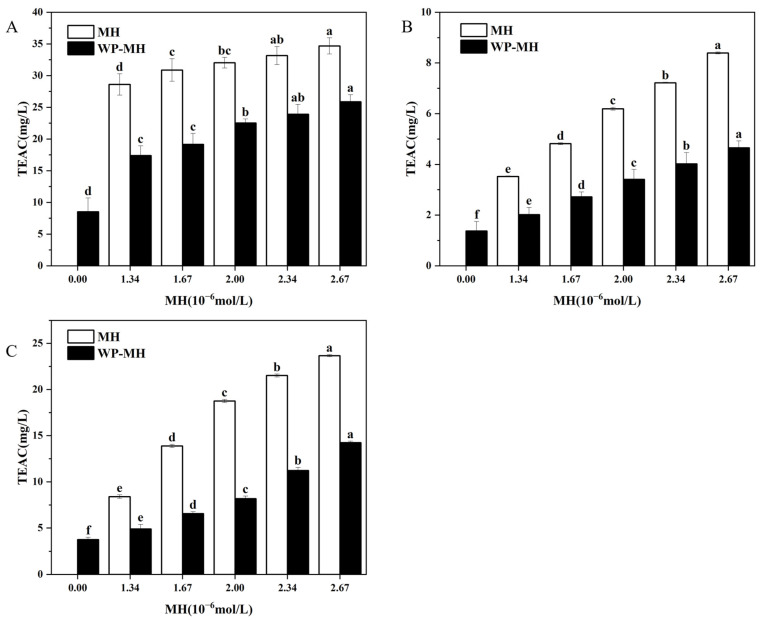
The antioxidant activity of the WP and WP-MH complex. (**A**) ABTS assay, (**B**) DPPH assay, and (**C**) FRAP assay. Different lowercase letters in the same color column represent significant differences (*p* < 0.05). The TEAC indicated on the *y*-axis of the figure is the Trolox equivalent antioxidant capacity.

Figure 5A shows that WP without MH has an ABTS clearance of 9.09 mg/L equivalent Trolox. Upon non-covalent binding with MH, the free radical scavenging ability of WP significantly increased to 17.39 mg/L (1.34 μM MH). And the scavenging capacity inclined to 25.88 mg/L at 2.67 Μm MH, suggesting that the bonded MH may contribute to the antioxidant properties of WP. As depicted in the molecular docking simulation, the high antioxidant MH was incorporated into the hydrophobic cavity, which may result in a higher capacity for WP to terminate the free radical chain reaction [28]. However, all complexes exhibited lower free radical scavenging ability than the corresponding free MH at all determined concentrations. The diminished antioxidant capacity for MH after binding to WP may be due to the interaction between the hydroxyl groups in MH and amino acids in WP, which not only reduced the number of free hydroxyl groups that make great contributions to its antioxidant activity but also confined its mobility [62].

Additionally, the ABTS free radical scavenging method has also been previously used by us to assess the antioxidant capacity of WP in the presence of MH’s structural analogues, such as hesperidin (HES) and neohesperidin (NH). The results indicated that as the concentration of these structural analogues increases, the antioxidant activity of the complexes showed an overall upward trend. Compared to WP-MH complexes, the enhanced antioxidant activity in WP-NH complexes was more significant, while that in the WP-hesperidin complexes was weaker. What is more, the antioxidant capacity of the WP-NH complex was nearly equal to that of free NH at fixed concentrations, while it was higher than that of the other two complexes. According to instances in the literature, the antioxidant capacity of protein-polyphenol complexes was greatly affected by the interaction between them, which was predominantly driven by hydrogen bonds and maintained by hydrophobic interactions. In this sense, the binding was closely related to the hydrophobicity of polyphenols, which largely depended on the structural characteristics of polyphenols, including the number and position of phenolic hydroxyl groups, glycosidic positions, and the spatial structure [63,64]. In the preliminary research, it was found that the binding strength between these polyphenols and WP was in the order of MH > HES > NH. The stronger affinity of MH to WP may be assigned to the methoxy groups, which increased hydrophobicity and enhanced the interaction between MH and WP. While the weaker binding for NH may be due to the β -1,2-glucoside structure, which improved the hydrophilicity of NH and diminished its binding affinity for WP. As a result, the phenolic hydroxyl groups of NH in the WP-NH complex had stronger degrees of freedom and exhibited stronger antioxidant activity [65]. In addition, WP-MH has a stronger antioxidant capacity than WP-HES, although the number of phenolic hydroxyl groups of MH is lower than that of HES. This may be due to the additional oxygen atoms in the MH molecule that help to form stable hydrogen bonds with WP, thus stabilizing the complex, and at the same time leaving some of the phenolic hydroxyl groups unbound from WP to play an antioxidant role. In contrast, the phenolic hydroxyl groups in HES may form hydrogen bonds with WP, spatially hindering its antioxidant portion and making it less active.

#### 3.7.2. DPPH Free Radical Scavenging Capacity

Figure 5B reveals the DPPH free radical scavenging activity of the WP alone was 1.37 mg/L. It progressively improved to 4.65 mg/L as the MH content reached 2.67 μM. This observation again confirmed that the incorporation of MH could enhance the antioxidant activity of WP. Similarly, the free radical scavenging ability of complexes was found to be lower than that of free MH, which was in line with the ABTS method. Meanwhile, the results also showed no difference compared with our previous reports, which involved the influence of MH’s structural analogues, such as HES and NH, on the antioxidant activity of WP [17,33]. Mechanistically, the DPPH method primarily relies on the ability of antioxidants to transfer DPPH· to DPPH [66]. Consequently, the DPPH scavenging activity of antioxidants is predominantly dependent on their hydrogen-donating capacity [67]. It may be the binding of MH with WP, consuming certain free phenol hydroxyl groups, that diminished its hydrogen-donating capacity, resulting in lower antioxidant levels than MH alone [68]. Furthermore, the residual free phenolic hydroxyl groups maintain their hydrogen-donating ability, and their quantity progressively increases with the elevation of MH concentration in the complex. This leads to the enhancement of the antioxidant properties of the complexes. In addition, the researchers reported that the free radical scavenging ability of β-LG (one important component in WP) was mainly attributed to changes in the location of Trp, Tyr, or Met residues in β-LG [69]. Liu et al. found that the enhanced antioxidant ability of the β-La/AST complex was partly attributed to the changes in the location of Met107 in β-La, which is involved in the binding interaction between AST and β-La [11]. Similarly, Tyr103 and Trp104 were found to participate in the interaction between HES and WP, while no binding was detected between NH/MH and Trp, Tyr, or Met residues. The different binding positions within β-La for NH, MH, and HES may be ascribed to their different structures. While the specific reasons for the increased antioxidant activity of WP after incorporation of MH need further investigation.

#### 3.7.3. FRAP-Reducing Power

The capacity of reducing potassium ferricyanide to potassium ferricyanide has been correlated to the antioxidant capacity and is widely used to evaluate the antioxidant activity of compounds. The influence of MH concentrations on the reducing capacity of MH-WP complexes was determined, as shown in Figure 5C. Clearly, the FRAP-reducing capacity of the WP control was 3.75 mg/L, and the values progressively increased along with increases in MH concentration. The results were consistent with the ABTS and DPPH assays. According to instances in the literature, the FRAP assay determines the ferric-reducing capacity, the ABTS assay measures the scavenging ABTS radical’s potential, and the DPPH assay assesses the hydrogen-donating capacity for scavenging DPPH radicals. It is not surprising that the reaction conditions and mechanisms of these methods are different, and the measured activity differs for these three methods. However, all methods confirm the enhancement of antioxidants after the binding of MH to WP. Additionally, although β-lactoglobulin and a-lactalbumin are both main components of WP, their enhanced antioxidant capacities may be different. The effects of epigallocatechin gallate on the antioxidant properties of five proteins were investigated by Almajano et al. [28]. It was found that the radical scavenging ability of the proteins after binding with EGCG was enhanced, and the antioxidant activity was in the following order: α-casein > β-casein > BSA > β-lactoglobulin > α-lactalbumin. Thus, which component makes a greater contribution to the enhanced antioxidant activity of WP-MH complexes needed to be further investigated.

## 4. Conclusions

In this work, the potential of enhancing the antioxidant activity of WP by interacting with MH was verified. The results indicated that MH could statically quench the intrinsic fluorescence of WP and alter the microenvironment of aromatic amino acid residues within WP. The interaction between MH and WP was a spontaneous endothermic reaction, with hydrophobic interaction being the main driving force, and they bonded together at a molar ratio of 1:1. The molecular docking simulation suggested MH entered into the hydrophobic cavity in WP and formed hydrogen bonds, van der Waals forces, hydrophobic interactions, etc., with amino acid residues. It may be the incorporation of MH that destroyed the ordered α-helix structures into unordered loose β-sheets and tightened the side chains of WP, which enhanced the antioxidant activity of WP in the end. Additionally, α-LA and β-LG are predominant components of WP, and their interaction with MH may be different. Further investigation is required to examine the effects of MH on the conformation and antioxidant ability of α-LA and β-LG. Moreover, the non-covalent forces maintained the binding between the protein and the MH, which was weak and susceptible to environmental factors, such as temperature, ionic strength, and pH, which in turn influenced the stability of the WPI-MH complex and its antioxidant capacity. Additionally, previous research has demonstrated that protein-polyphenol interactions impact both the digestibility of proteins and the bioavailability of polyphenols. Therefore, it is crucial to conduct further related experiments to gain a comprehensive understanding of the impact of MH on WPI. This could provide valuable insights for designing effective antioxidant products and their applications in the food and medical fields.

## Figures and Tables

**Table 1:** The quenching constant (K_sv_), binding constants (K_a_), and binding site number (n) for the interaction of WP with MH at different temperatures.

T (K)	K_sv_ (10^5^ M^−1^)	K_q_ (10^12^ M^−1^ S^−1^)	R^a^	n	K_a_ (10^4^ M^−1^)	R^b^
298	0.50 ± 0.01 ^a^	4.98 ± 0.01 ^a^	0.998	1.038 ± 0.0198 ^a^	7.16 ± 0.095 ^a^	0.998
304	0.49 ± 0.02 ^b^	4.89 ± 0.02 ^b^	0.994	1.023 ± 0.0330 ^a^	5.56 ± 0.159 ^b^	0.995
310	0.40 ± 0.02 ^c^	4.02 ± 0.02 ^c^	0.992	1.035 ± 0.0405 ^a^	5.45 ± 0.195 ^b^	0.992

R^a^ is the correlation coefficient for the K_sv_ values. R^b^ is the correlation coefficient for the K_a_ values. Different lowercase letters in the same column represent significant differences (*p* < 0.05).

**Table 2:** The thermodynamic parameters for the interaction of WP with MH at different temperatures.

T (K)	ΔH (KJ/mol)	ΔG (KJ/mol)	ΔS (J/mol/K)
298	25.40 ± 0.129	−26.29 ± 0.15 ^a^	173.46 ± 0.95
304		−27.33 ± 0.12 ^b^	
310		−28.38 ± 0.19 ^c^	

Different lowercase letters in the same column represent significant differences (*p* < 0.05).

**Table 3:** The contents of secondary structures of WP in the presence of increasing amounts of MH; the mass ratios of WP to MH were 1:0, 2:1, 1:1, 1:2, and 1:4.

WP-MH	α-Helix (%)	β-Sheet (%)	β-Turn (%)	Random Coil (%)
1:0	18.8	31.1	19.2	30.5
2:1	8.1	52.7	17.3	28.0
1:1	7.0	52.7	17.0	28.6
1:2	6.7	52.9	16.8	28.6
1:4	6.4	52.9	16.7	28.8

## Data Availability

The original contributions presented in this study are included in the article. Further inquiries can be directed to the corresponding author(s).

## Abbreviations

The following abbreviations are used in this manuscript:

WP	Whey protein
MH	Methyl hesperidin
HES	Hesperidin
NH	Neohesperidin
CD	Circular Dichroism
ABTS	2,2′-Azinobis-(3-ethylbenzothiazoline-6-sulfonate)
DPPH	1,1-diphenyl-2-picrylhydrazyl
FRAP	Ferric ion reducing antioxidant power
TPTZ	2,4,6-Tri(2-pyridyl)-s-triazine
Trp	Tryptophan
Tyr	Tyrosine
α-LA	α-Lactalbumin
β-LG.	β-Lactoglobulin
TEAC	Trolox equivalent antioxidant Capacity
